# Expression of an alternatively spliced variant of *SORL1* in neuronal dendrites is decreased in patients with Alzheimer’s disease

**DOI:** 10.1186/s40478-021-01140-7

**Published:** 2021-03-16

**Authors:** Giulia Monti, Mads Kjolby, Anne Mette G. Jensen, Mariet Allen, Juliane Reiche, Peter L. Møller, Raquel Comaposada-Baró, Bartlomiej E. Zolkowski, Cármen Vieira, Margarita Melnikova Jørgensen, Ida E. Holm, Paul N. Valdmanis, Niels Wellner, Christian B. Vægter, Sarah J. Lincoln, Anders Nykjær, Nilüfer Ertekin-Taner, Jessica E. Young, Mette Nyegaard, Olav M. Andersen

**Affiliations:** 1 Department of Biomedicine, Aarhus University, Høegh-Guldbergs Gade 10, 8000 Aarhus C, Denmark; 2 Department of Neuroscience, Mayo Clinic, Jacksonville, FL 32224 USA; 3 Department of Biochemistry, Jena University Hospital, Jena, Germany; 4 Department of Clinical Medicine, Aarhus University, Aarhus C, Denmark; 5 Department of Pathology, Randers Regional Hospital, Randers, Denmark; 6 Department of Medicine, Division of Medical Genetics, University of Washington, Seattle, WA USA; 7 Center for Proteins in Memory - PROMEMO, Danish National Research Foundation, Department of Biomedicine, Aarhus University, Aarhus C, Denmark; 8 Department of Neurology, Mayo Clinic, Jacksonville, FL 32224 USA; 9 Department of Pathology, Institute for Stem Cell and Regenerative Medicine, University of Washington, Seattle, WA USA

**Keywords:** Alzheimer’s disease, SORLA, *SORL1*, Alternative splicing, Dendritic transcript

## Abstract

*SORL1* is strongly associated with both sporadic and familial forms of Alzheimer’s disease (AD), but a lack of information about alternatively spliced transcripts currently limits our understanding of the role of *SORL1* in AD. Here, we describe a *SORL1* transcript (*SORL1-38b*) characterized by inclusion of a novel exon (E38b) that encodes a truncated protein. We identified E38b-containing transcripts in several brain regions, with the highest expression in the cerebellum and showed that *SORL1-38b* is largely located in neuronal dendrites, which is in contrast to the somatic distribution of transcripts encoding the full-length SORLA protein (*SORL1-fl*). *SORL1-38b* transcript levels were significantly reduced in AD cerebellum in three independent cohorts of postmortem brains, whereas no changes were observed for *SORL1-fl*. A trend of lower 38b transcript level in cerebellum was found for individuals carrying the risk variant at rs2282649 (known as SNP24), although not reaching statistical significance. These findings suggest synaptic functions for *SORL1-38b* in the brain, uncovering novel aspects of *SORL1* that can be further explored in AD research.

**Supplementary Information:**

The online version contains supplementary material available at 10.1186/s40478-021-01140-7.

## Introduction

The *SORL1* gene encodes the protein SORLA and is associated with Alzheimer’s disease (AD) [1]. Recent burden analyses of ultra-rare variants through exome sequencing have found an excess of loss-of-function variants in AD cases, suggesting haploinsufficiency of *SORL1* as a pathogenic mechanism in some patients [17, 44]. More recently, *SORL1* missense variants in familial AD have also been reported [11, 29, 32], but the functional consequences of these variants are unknown.

Previous studies have established SORLA as a sorting receptor for the amyloid precursor protein (APP) [1, 2]. This function depends on the physical contact between the extracellular parts of SORLA and APP, and the ability of the cytoplasmic tail of SORLA to form complexes with intracellular trafficking molecules including the retromer complex [15, 39]. Apart from its role as an APP trafficking determinant, very limited information exists about SORLA neuronal functions. There is therefore an increasing need to better understand the function of SORLA in the brain.

Alternative splicing (AS) is an essential process significantly involved in the expansion of the transcriptome and protein diversity. As another result of AS, transcripts from the same gene can also have different 3′ untranslated regions (3′ UTRs) and/or contain target motifs for RNA binding proteins within exonic sequences, responsible for distinct neuronal trafficking of transcript isoforms to axons or dendrites where they may be locally translated [12, 24, 43]. Recently, emerging evidence links AS with the maintenance of neuronal homeostasis [34], and associations between AS and AD have been reported [35]. For this reason, increased attention is directed towards AS of genes involved in neurodegenerative and neuropsychiatric diseases.

Although transcripts from ~ 95% of all human multi-exon genes undergo AS [31], surprisingly little is known about the biological relevance for AS of *SORL1*. Due to an increasing number of putative transcripts annotated in databases, it is key to conduct experimental investigations to discriminate between functional transcripts and products derived by transcriptional noise. Here, we provide a detailed characterization of an alternatively spliced *SORL1* transcript. Inclusion of a hitherto undescribed exon leads to transcripts that can be translated into a truncated receptor lacking its transmembrane and cytoplasmic domains, pointing towards a function unrelated to sorting of cargoes including APP. Using brain samples from three independent cohorts, we found decreased transcript expression of this truncating *SORL1* isoform in AD patients, and identified enriched expression in neuronal dendrites suggesting a role of this novel isoform in synaptic plasticity, known to be impaired in AD.

## Materials and methods

### Human samples

We used biological material from four different sources: total RNA acquired from ClonTech, and human postmortem brain tissues from three different brain banks; the Netherlands Brain Bank (NBB), Mayo Clinic (Mayo), and University of Washington (UW).

*From ClonTech,* we obtained total RNA from brain, spinal cord, bone marrow, liver, heart, lung, trachea, kidney, adrenal gland, salivary gland, thyroid gland, thymus, skeletal muscle, colon, prostate, testis, placenta, and uterus.

*From NBB*, postmortem cerebella (n = 3 AD; n = 3 non-AD), hippocampus (n = 3 non-AD) and entorhinal cortex (n = 3 non-AD) samples were obtained for in situ hybridization (ISH) (Table 1). NBB procedures have been approved by the ethics committee at the Vrije University Medical Center, Amsterdam, NL. The materials were donated to the bank on the basis of signed informed consent with the restriction of research purposes only. Diagnosis of AD was made by neuropsychological testing, followed by autoptic histopathological confirmation with disease propagation described by Braak staging. Following autopsy, cerebellum was dissected at the level of dentate nucleus, fixed in 4% paraformaldehyde and the tissue was embedded in paraffin blocks.

**Table 1.** Netherlands Brain Bank samples data

Region	Diagnosis	Sex	Age	PMD	pH	Weight	ApoE	Braak stage
Cerebellum	Non-AD	M	72	07:25	6.61	1370	3/3	0
Cerebellum	Non-AD	M	69	05:55	6.41	1450	3/3	0
Cerebellum	Non-AD	M	62	09:35	6.58	1163	3/3	0
Cerebellum	AD	M	75	04:20	6.91	1325	3/3	6
Cerebellum	AD	M	58	05:10	6.99	1180	3/3	6
Cerebellum	AD	M	69	05:30	6.18	1086	3/3	6
Hippocampus	Non-AD	M	91	08:00	6.26	1188	3/3	1
Hippocampus	Non-AD	M	79	05:45	6.38	1361	3/3	2
Hippocampus	Non-AD	M	96	04:10	6.09	1250	3/3	4
Amygdala/EC	Non-AD	M	81	07:55	6.23	1194	3/3	2
Amygdala/EC	Non-AD	M	78	< 17:40	6.52	1125	3/3	1
Amygdala/EC	Non-AD	M	79	05:45	6.38	1361	3/3	2

*PMD* post-mortem delay

*From Mayo Clinic*, postmortem brain samples (n = 25 AD; n = 25 non-AD) were obtained for qPCR as previously described [5] and eQTL analysis (Table 2). Briefly, non-AD control samples had a Braak score of 3.0 or less and lacked any other major pathologic diagnoses; AD patients had a Braak score of ≥ 4.0 and a definite diagnosis according to the NINCDS-ADRDA criteria [27]. Individuals were age matched across diagnosis groups (± 1 year), were older than 60 years at time of death and 48% of each diagnosis group were male. This study was approved by the appropriate institutional review board.

**Table 2:** Mayo Clinic cohort data

Sample id	Diagnosis	Age at death	Gender	RIN	GENOTYPE_rs2282649
14546_CER	Non-AD	83	M	8.3	C/C
14548_CER	Non-AD	83	M	8.5	C/C
14549_CER	Non-AD	63	M	8.8	C/C
14551_CER	Non-AD	80	F	9.3	C/C
14556_CER	Non-AD	72	F	8.9	C/T
7101_CER	Non-AD	79	M	7.3	na
7104_CER	Non-AD	78	F	8.6	na
14543_CER	Non-AD	80	M	6.6	C/C
14562_CER	Non-AD	76	M	8.1	C/T
1926_CER	Non-AD	88	F	7.7	C/C
1936_CER	Non-AD	89	F	6.6	C/T
1955_CER	Non-AD	87	F	8.3	C/C
1960_CER	Non-AD	97	F	7.6	C/C
1963_CER	Non-AD	94	M	9.1	C/T
1964_CER	Non-AD	92	M	9.2	C/C
14557_CER	Non-AD	81	M	8.1	C/T
1919_CER	Non-AD	87	F	9.2	C/T
1933_CER	Non-AD	98	F	7.8	C/C
1934_CER	Non-AD	89	M	9.7	C/T
1937_CER	Non-AD	86	F	8.5	C/C
1940_CER	Non-AD	96	M	7.5	C/C
1944_CER	Non-AD	98	F	7.1	C/C
1945_CER	Non-AD	90	M	8.9	C/C
1950_CER	Non-AD	86	F	7.6	C/T
1952_CER	Non-AD	92	F	8.8	C/T
1058_CER	AD	95	M	7.5	C/C
1166_CER	AD	84	M	8.5	C/T
142_CER	AD	89	F	9.2	C/C
747_CER	AD	62	M	8.9	na
807_CER	AD	81	M	7.9	na
851_CER	AD	87	F	9.1	C/T
896_CER	AD	80	F	9.1	C/C
1034_CER	AD	88	F	7.8	T/T
1046_CER	AD	72	F	9.2	C/C
1052_CER	AD	91	M	8.4	C/T
1114_CER	AD	82	M	8.4	T/T
1169_CER	AD	92	M	8.5	na
907_CER	AD	95	F	7.6	na
953_CER	AD	86	F	8.3	na
720_CER	AD	75	M	8.1	na
741_CER	AD	89	M	8.4	na
790_CER	AD	86	F	7.5	na
809_CER	AD	87	F	8.4	na
911_CER	AD	89	F	7.3	na
963_CER	AD	99	F	7.7	na
990_CER	AD	79	M	6.7	na
1101_CER	AD	78	M	7.6	na
1146_CER	AD	100	F	8.5	C/C
731_CER	AD	93	M	9.0	T/T
966_CER	AD	79	2	8.8	C/T

*RIN* RNA integrity number

*From UW*, postmortem cerebella tissue (n = 14 AD; n = 6 non-AD) were selected for qPCR based on SNP24 genotype (Table 3).

**Table 3:** University of Washington cohort data

Sample id	Diagnosis	Age at death	Gender	GENOTYPE_rs2282649
1706_CER	Non-AD	76	F	T/T
1661_CER	Non-AD	92	F	C/T
1776_CER	Non-AD	98	F	C/T
1750_CER	Non-AD	78	M	C/C
1792_CER	Non-AD	93	F	C/C
1814_CER	Non-AD	86	F	C/C
1798_CER	AD	93	F	T/T
1744_CER	AD	93	F	T/T
1745_CER	AD	82	F	T/T
1784_CER	AD	90+	F	T/T
1785_CER	AD	93	M	T/T
1815_CER	AD	67	F	T/T
1682_CER	AD	100	F	C/T
1733_CER	AD	92	F	C/T
1746_CER	AD	69	M	C/T
1771_CER	AD	98	F	C/T
1806_CER	AD	65	M	C/T
1662_CER	AD	82	F	C/C
1681_CER	AD	74	F	C/C
1707_CER	AD	90	M	C/C
1804_CER	AD	101	F	C/C

### *SORL1-38b* transcript levels across different human tissues

Total RNA from ClonTech was converted to cDNA using 1 μg of RNA and High Capacity RNA-to-cDNA kit (Cat N. #4387406, Applied Biosystems, Europe). PCR was performed using Herculase II Fusion DNA Polymerase (Cat N. #600675, Agilent, Europe) on a Veriti Thermal Cycler (Applied Biosystems) in the presence of 3% DMSO with the following optimized protocol: 95 °C for 2 min, followed by 35 cycles of amplification (95 °C for 20 s, 52 °C for 20 s and 72 °C for 30 s) and extension at 72 °C for 3 min. *SORL1-38B* was amplified using primers in exon 35 and 38b. Primer sequences were SORL1-Ex35-fw: 5′-GGCACACAACACCAATGACT, and SORL1-Ex38B-rev; 5′-TGCTCTTCCAACATCCCTTCT. Water was used as negative control. RT-PCR for the analysis of splicing events downstream of E38b was performed using Titanium One-Step RT-PCR Kit (Cat N. 639504, ClonTech) on total RNA from human cerebellum (Cat N. 636535, ClonTech) with primers located in exons 38b and 40. Primer were SORL1-Ex38b-fw: 5′-TGACCACACATACCAAGAAGGG and SORL1-Ex40-rev: 5′-TGCTTCCTCGGAAGTTCAAAGT. The refined analysis of transcript levels across tissue was performed using qPCR on the same samples and protocol for cDNA synthesis. Each cDNA was tested with a custom Taqman probe for detection of *SORL1-38b* (SORLA_EX_37-38B, AssayID AJBJXYH, Cat N. 4441114) (Applied Biosystems), and pre-designed probes for *SORL1 exon 3–4* (Hs00268342_m1). Three household genes were analysed: *HPRT1* (Hs02800695_m1), *TFRC* (Hs00951083_m1), and *TXNL1* (Hs00355488_m1). Singleplex PCR was performed in 96-well plates LightCycler 480 System (Roche) with the following conditions: 95 °C for 10 s, 45 cycles of amplification (95 °C for 10 s, 60 °C for 30 s, 72 °C for 1 s), and 72 °C for 1 s. The efficiency and dynamic range for each qPCR assay was established using a serial dilution, and confirmed to be above 92% for all assays.

### Generation of *SORL1-38b* expression construct

The small fragment in human *SORL1* from exon 37 to exon 38B was synthesized in pEX-A2 vector (MWG, Eurofins, Germany) including the native NdeI site in exon 37 and a NsiI site at the 3′ end. The fragment was cloned using NdeI and NsiI into the human *SORL1* in pGEM EASY vector [19], and was subsequently subcloned to a pcDNA3.1 (Zeo) vector for cell studies. Site directed mutagenesis was used for introducing mutations leading to Cys to Ala substitutions at position 1 and 13 of the tail encoded by E38b using Quickchange XL Site-Directed Mutagenesis (Cat N. 200516) (Agilent). The same approach was applied for introducing stop codons after each 3Fn domain in a pcDNA3.1 plasmid containing the cDNA for human *SORL1*.

### Antibodies

A polyclonal antibody against SORLA-38b was generated in rabbit by peptide immunization against the unique c-terminal VCFKTLTTHTKKGCWKSNQIHVP encoded by E38b (Aldevron, Germany). The immunogen CFKTLTTHTKKG-Abu-WKSNQIHVP was synthesized, where the C-terminal cysteine was substituted with aminobutyric acid (Abu). Keyhole limpet hemocyanin was conjugated to the immunogen for immunization. The antibody was subsequently purified from serum, and its specificity was assessed by Western blot (WB) analysis.

Mouse monoclonal LR11 antibody was purchased from R&D systems (Cat N. 611860), and rabbit polyclonal anti-sol SORLA (IgG 5387) and anti-tail SORLA were from colleagues at Aarhus University.

### Cell culture

HEK-293 cells were cultured in Dulbecco’s Modified Eagle’s Medium (DMEM, Sigma) supplemented with 10% fetal bovine serum and penicillin/streptomycin (P/S) in a humidified 5% CO_2_ incubator at 37 °C. Fugene 6 Transfection Reagent kit (Cat N. E2691, Promega) was used to transfect cells with *SORL1-38b* or *SORL1-fl* constructs according to manufacturer’s instructions. After 24 h, medium with zeocin was added to the transfected cells for selection and generation of a stable cell line expressing *SORL1-38b* construct. CHO cells were cultured in HyClone medium (Sigma) supplemented with 5% P/S, and were transfected with *SORL1* constructs using Fugene kit.

### Pulse-chase analysis

Cells were incubated in methionine- and cysteine-free medium (Cat N. D0422, Sigma) supplemented with glutamax and 2% dialyzed FBS (Gibco) for 15 min at 37 °C, prior to biolabeling with [^35^S] Promix (Amersham Biosciences) for 40 min at 37 °C. Subsequently, cells were washed with phosphate-buffered saline (PBS), and both media and lysates were collected at different chase points. Cell lysates were immunoprecipitated at 4 °C overnight with GammaBind G-Sepharose beads (Amersham Biosciences) previously labeled with anti-sol-SorLA diluted in HyClone medium. Beads were then washed with PBS and proteins were resolved by standard SDS-PAGE and fluorography.

### Immunoblotting

Transfected cells were lysed in lysis buffer containing protease inhibitor (Complete, Roche), and media were collected. Proteins were fractioned by 4–12% SDS-gel electrophoresis, and transferred to nitrocellulose membranes (Amersham). Membranes were blocked in blocking buffer (20% TST buffer (0.25 M Tris-Base, 2.5 M NaCl, 0.5% Tween 20 pH 9), 2% skimmed milk powder, 2% Tween 20) for 1 h at RT, and incubated at 4 °C overnight with mouse anti-LR11 (1:500), and rabbit pAb-38b (1:1000) primary antibodies. Incubation with HRP-conjugated anti-mouse (1:1500) or anti-rabbit (1:1500) secondary antibodies was performed at RT for 1 h. Proteins were detected with SuperSignal West Femto Maximum Sensitivity Substrate (Thermo Fisher) and visualized with LAS-1000 (GE Healthcare).

### In situ hybridization

From NBB, paraffin embedded human cerebellum, hippocampus and entorhinal cortex were cut to 5 μm thick sections using a microtome. ISH was carried out by the BaseScope and BaseScope Duplex technologies (Advanced Cell Diagnostics) following manufacturer’s instructions. Tissue hybridization was performed for 2 h at 40 °C (Hybez oven, ACDbio) with specific probes designed to span exon-exon junctions for recognition of *SORL1-38b* (E38E38b; E38bE39; E38bE40) and *SORL1-fl* transcripts (E38E39). In parallel, sections were hybridized with a PPIB probe (Cat N. 322975, ACDBio) as positive control. Chromogenic detection of specific hybridization was assessed with Fast Red dye (Cat N. 323600, ACDbio), and Fast Red-Green (Cat N. 323800, ACDBio). Nuclei counterstaining was made by incubation with Mayer’s hematoxylin for 2 min at RT. Tissues were rinsed in deionized water and mounted with VectaMount permanent medium (Vector labs, Burlingame, CA). Slices were imaged with a Zeiss Apotome brightfield camera (Zeiss Apotome). Number of transcripts was manually quantified by counting punctate dots observed in PCs in the entire sections. Data were then plotted as mean number of transcripts per counting group (Group 0: 0 signal; Group I: 1 signal; Group II: 2–3 signals; Group III: > 4 signals). For quantification of somatic and extra-somatic transcripts after BaseScope treatment, 25 random fields were chosen at 20X magnification, counting three PCs in each field in order to compare homogenous tissue areas.

### RNA-seq read alignment

Two published datasets were queried for the presence of RNA-seq reads spanning the exon 38b splice junctions. We downloaded RNA sequencing alignment files generated from the cerebellum [33] along with frontal lobe, superior temporal gyrus and parahippocampal gyrus brain regions generated from the Mount Sinai Brain Bank [46]. We quantified the ratio of E38E38b versus E38E39 splice junctions reads. Reads spanning the E38E38b, E38bE39 and E38bE40 junctions collected from parahippocampal gyrus samples were overlaid onto the UCSC human genome browser.

### *SORL1-38b* differential expression in AD and non-AD samples

The cDNA synthesis and qPCR analysis on the Mayo samples were conducted at Mayo Clinic. RNA was isolated from 50 cerebellum samples from the Mayo Clinic brain bank using TRIzol Reagent (Ambion Life Technology) followed by DNase RNA cleanup step using RNeasy (Qiagen). The quantity and quality of RNA samples were determined by the Agilent 2100 Bioanalyzer using Agilent RNA 6000 Nano Chip. All samples had an RNA integrity number (RIN) of ≥ 6.6, with an equivalent mean RIN for both diagnosis groups (mean RIN AD = 8.26, mean RIN controls = 8.24). cDNA synthesis was performed with 1–2 μg of RNA using the High Capacity RNA-to-cDNA kit (Applied Biosystems cat. no. 4387406) according to the manufacturer’s instructions. The samples were tested with a custom Taqman probe for detection of *SORL1-38b*, and three housekeeping genes: *HPRT1*, *TFRC*, and *TXNL1*. Four replicate measures were taken per sample for each of the four Taqman assays (16 measures per sample) on 384 well reaction plates using the QuantStudio™ 7 Flex Real-Time PCR system and analysis software (Applied Biosystems, California, USA).

The cDNA synthesis and qPCR analysis on the UW samples were conducted at the University of Washington. From each sample, 1 μg total cerebellum RNA was converted to cDNA (iScript, Biorad). SYBR Green qPCR was performed using Real-Time PCR Detection System (Biorad) with the following primers for *SORL1-38b*: E38b-Fw: 5′-GCGGTGACTAGTCGTGGAAT and E38b-Rev: 5′-TGCTCTTCCAACATCCCTTC. PCR products were amplified with the following conditions: 50 °C for 2 min, 95 °C for 20 s, 40 cycles of amplification (95 °C for 3 s, 60 °C for 30 s). Three housekeeping genes were used as controls: *HPRT1*, *CYC1* and *RPL13*.

Each sample was run in triplicates, and water was included as negative control.

### Genotyping of SNP24

Briefly, genomic DNA was prepared from cerebella samples from UW Neuropathology Core. DNA was genotyped for the presence of risk (T) or protective (C) allele at position rs2282649 by Sanger Sequencing (Genewiz). In total, 67 samples were genotyped and we observed 7 T/T carriers (10.4%), 29 C/C carriers (43.3%), and 31 C/T carriers (46.3%). To obtain groups of equal sample size, we chose 7 samples of each genotype (C/C, C/T, and TT) for 38b transcript analysis. One sample from the T/T genotype group failed qPCR of *SORL1-38b* due to Ct values outside the dynamic range for the assay. Primers for PCR of amplicon containing SNP24 was FW: 5′-CTTTAGCTCATTCAGTATTCTTACTGTATG, and Rev: 5′-GAGCATTTCTTCTAATGCAGACATAC.

### Statistical analysis

#### In situ semi quantitative analysis

An unpaired student's t test was performed in Graphpad Prism 5.0 to compare differences and determine statistical significance between AD and non-AD samples. A *p* value below 0.05 was considered significant, indicated as *p* < 0.05 (*), *p* < 0.01 (**), and *p* < 0.001 (***).

#### Differential expression of 38b transcript in AD versus non-AD

Comparison of normalized *SORL1-38b* transcript levels between AD and non-AD samples in the Mayo and UW cohorts was performed in R version 3.6.1. First, a linear regression between deltaCT and diagnosis was performed, with RIN (only available for Mayo cohort), age at death, gender and plate ID as covariates. As no significant association was found for any of the covariates, a Wilcoxon rank sum test was subsequently used to compare AD versus non-AD samples. A meta-analysis of the two cohorts was performed using the metap R package (version 1.3). (Dewey M (2020). metap: meta-analysis of significance values*).*

#### Association of SNP24 with AD

Comparison of the risk allele frequency between AD and non-AD samples for SNP24 in the Mayo cohort was performed using Fisher’s exact test. This test was not performed in the UW cohort, as samples were chosen based on genotype, so allele frequencies were not representative for AD cases and controls.

#### Expression of 38b transcript stratified on SNP genotype

Whole genome-sequence data for 35 of the Mayo Clinic samples is available via the AD Knowledge Portal (https://adknowledgeportal.synapse.org, 10.7303/syn2580853). The AD Knowledge Portal is a platform for accessing data, analyses, and tools generated by the Accelerating Medicines Partnership (AMP-AD) Target Discovery Program and other National Institute on Aging (NIA)-supported programs to enable open-science practices and accelerate translational learning. The data, analyses and tools are shared early in the research cycle without a publication embargo on secondary use. Data is available for general research use according to the following requirements for data access and data attribution (https://adknowledgeportal.synapse.org/DataAccess/Instructions). For access to content described in this manuscript see: https://www.synapse.org/#!Synapse:syn11714444. Genotypes were extracted from the available VCF files using vcftools version 0.1.17.

To determine the effect of SNP24 on transcript levels, type I ANOVA was used to compare the three groups, C/C, C/T and T/T, with diagnosis as covariate. Normal distribution was checked visually using qqplot and Shapiro–Wilk test. The eQTL analysis of 38b was performed on 35 samples from the Mayo cohort using the MatrixEQTL package [41]. The cis-eQTL analysis was restricted to SNPs in the *SORL1* gene ± 1 Mbp. Only SNPs with at least 5 and at most 65 alternate allele counts of the total 70 alleles were used. MatrixEQTL was run with modelLINEAR and disease status as covariate.

## Results

### Identification of a novel primate-specific exon in SORL1

The *SORL1* gene on human chromosome 11 spans > 181 kb and contains at least 48 assigned exons [36]. The mRNA produced by assembling these known exons encodes the main form of SORLA referred to as the full-length receptor (SORLA-fl).

**Fig. 1:**
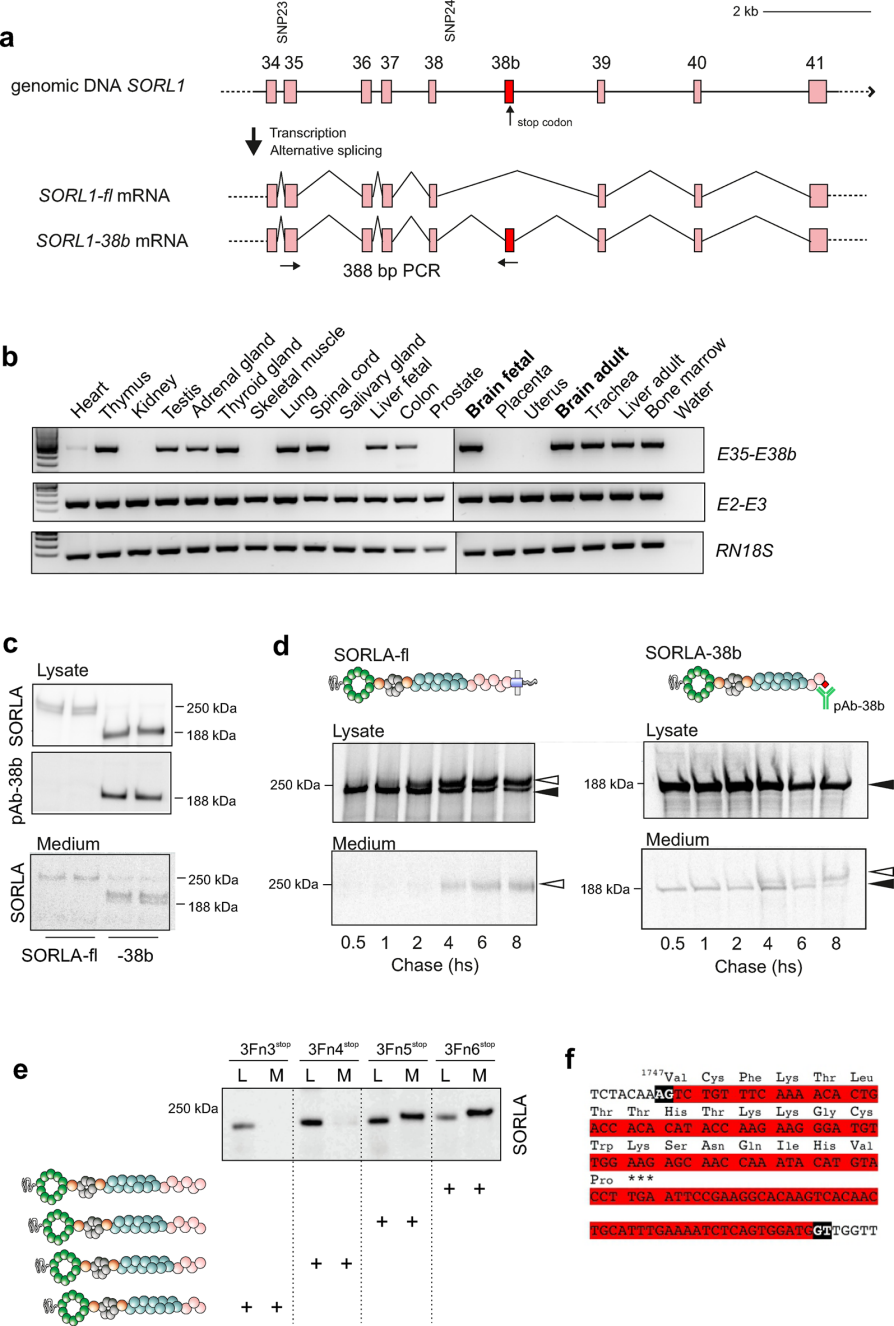
A novel alternatively spliced *SORL1* transcript encodes a truncated receptor. **a** Schematic representation of the genomic region of *SORL1* including the novel exon 38b (red) located in close proximity to SNP24 within the 3′ risk haplotype (~ 1200 bp). Primers for amplification of the 388 bp fragment between exon 35 and 38b are indicated with arrows. **b** RT-PCR showing specific expression of *SORL1-38b* transcripts (E35–E38b) in only some human tissues, compared to ubiquitous expression of *SORL1* transcripts containing exons 2 and 3 (E2–E3), and 18S ribosomal RNA (RN18S) as control. **c** WB analysis of lysates and media of cells transfected with cDNA encoding either SORLA-fl or SORLA-38b. The antibody pAb-38b specifically recognizes the truncated 188 kDa SORLA-38b receptor, but not the SORLA-fl protein (250 kDa). **d** [^35^S]-pulse-chase analysis on cells transfected with constructs encoding SORLA-fl (left) or SORLA-38b (right), with both isoforms mainly located in cell lysates. SORLA-fl goes from an immature (black arrowhead) to a mature (white arrowhead) form during the 8 h chase period, and the mature protein can be shed into the medium after 4 h. Trace amounts of SORLA-38b is secreted as early as 30 min after radiolabeling, but can undergo endocytosis and be re-secreted in a mature form after 4 h. **e** WB analysis for a panel of SORLA deletion constructs terminating after different 3Fn-domains in cell lysates (L) or medium (M), showing how the presence of the fifth 3Fn-domain (3Fn5) is responsible for cellular retention of soluble SORLA constructs. **f** Genomic sequence of E38b (in red) with flanking splice regions (in black), including translated amino acid sequence shown on top

Here we validated the existence of a novel 118 bp exon located between exons 38 and 39, hereafter referred to as “exon 38b” (E38b) (Fig. 1a). A human tissue RNA library was screened by RT-PCR using an E38b specific primer pair (for exons 35/38b), revealing inclusion of this exon in transcripts from numerous tissues, including both fetal and adult brain. Several tissues did not express *SORL1-38b* although they were positive for canonical *SORL1* transcripts (identified by primer pair for exons 2/3), indicating differential expression of the alternatively spliced transcript (Fig. 1b).

Sequencing of the RT-PCR product from brain identified a E38b-containing transcript encoding a protein with 22 new amino acids followed by a stop-codon (Fig. 1f). The resulting truncated receptor protein is hereafter termed SORLA-38b (Fig. 1c). Notably, the junction between exons 38 and 39 corresponds exactly to the boundary between the second and the third fibronectin-type III (3Fn)-domains of SORLA (between residues 1747 and 1748). Alignment analysis of the genomic sequence across species confirmed E38b conservation from human to *Pan Troglodytes*, *Macaca mulatta*, and *Gorilla gorilla* genomes, whereas this exonic cassette is absent in non-primate phylogeny.

The presence of the unique 22 residues allowed us to raise an antibody (pAb-38b, Fig. 1c, d) specifically recognizing the unique C-terminal end of SORLA-38b, thereby enabling us to distinguish the truncated isoform from SORLA-fl, as demonstrated by WB analysis. As expected, only cells transfected with a cDNA for *SORL1-38b*, but not *SORL1-fl*, produced a product recognized by pAb-38b. Surprisingly, in this experiment only a limited fraction of SORLA-38b was secreted into the medium despite the lack of any transmembrane region, suggesting another mechanism of cell retention (Fig. 1c). A [^35^S]-pulse-chase experiment confirmed that SORLA-38b stays mainly cell-associated (Fig. 1d).

To investigate why SORLA-38b is not efficiently secreted, we experimentally substituted each of two cysteine residues that are present in the unique SORLA-38b sequence. We found that neither of the two residues—when mutated separately or simultaneously—had any effect on secretion (Additional file 1: Fig. S1a). As deletion of all 22 amino acids from the SORLA-38b isoform also did not affect secretion, we finally measured secretion of a panel of SORLA deletion constructs, demonstrating that the presence of the fifth 3Fn-domain is essential for secretion of the SORLA extracellular domain (Fig. 1e, Additional file 1: Fig. S1b). Although the exact molecular mechanism remains to be determined in detail, these data strongly suggest that the truncated SORLA-38b is structurally different compared to the full-length SORLA receptor, in line with distinct unrelated functions.

Combined, these findings indicate tissue-specific expression of a novel *SORL1* transcript, that can be translated into a truncated and stable SORLA-38b protein isoform with a function unrelated to the full-length receptor.

### High SORL1-38b expression in cerebellum

**Fig. 2:**
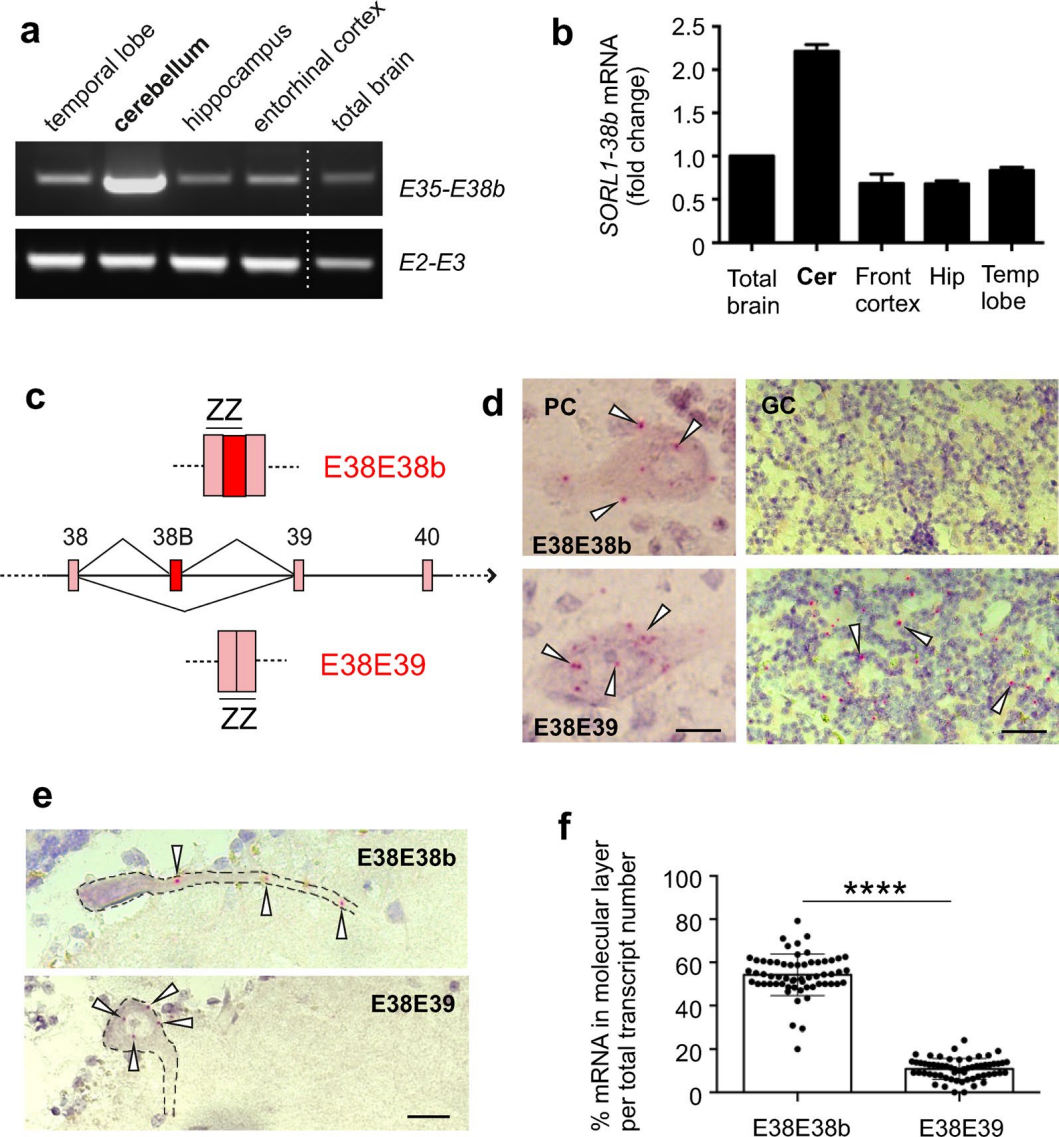
*SORL1-38b* is expressed in cerebellar Purkinje cells. **a** RT-PCR analysis showing expression of *SORL1-38b* (primers E35–E38b) and control *SORL1* transcripts (primers E2–E3) in brain regions. **b** qPCR analysis of *SORL1-38b* expression in human brain regions relative to housekeeping genes *HPRT1*, *TFRC* and *TXNL1*. Data are depicted as fold change relative to expression in total brain. *Cer* cerebellum, *Front cortex* frontal cortex, *Hip* hippocampus, *Temp lobe* temporal lobe. **c** Schematic representation showing how custom-designed BaseScope double-Z probes target the junctions between exon 38 and 38b (E38E38b), and between exon 38 and 39 (E38E39) to specifically detect E38b-containing transcripts. **d** BaseScope analysis using E38E38b probe confirms the presence of exon 38b-containing transcripts in human cerebellum. *PC* Purkinje cells, *GC* granule cells. Scale bar, 20 μm. **e** BaseScope images showing that E38E39 probe is mainly localized in the soma of PCs (bottom panel), whereas E38E38b probe signal is found in the soma but also distributed in the dendrites of PCs (top panel). Scale bar, 50 μm. **f** Quantification of the number of *SORL1-38b* (E38E38b) and *SORL1-fl* (E38E39) transcripts localized in the dendrites of PCs. Individual data points represent quantification from 3 independent brains, and in each section 25 random pictures showing homologous cerebellum regions were used for the analysis (n = 225 cells/probe). ****p < 0.0001

We next measured the expression levels of *SORL1-38b* across multiple brain regions by RT-PCR. While expression was evident for all tested regions including the temporal lobe, hippocampus, frontal cortex and entorhinal cortex, a consistently higher expression was observed in the cerebellum, a region previously reported to express high levels of SORLA [5, 28, 30]. A follow-up qPCR analysis demonstrated 2.1-fold enrichment of *SORL1-38b* transcript in cerebellum compared to whole brain (i.e. ΔΔCt = − 1.1) (Fig. 2a, b). Accordingly, we focused our first analysis on cerebellum.

To determine in detail the histological distribution of *SORL1-38b* transcript, we employed BaseScope technology for the in situ detection of the splice variant and *SORL1-fl* in brain specimens (Fig. 2c). Whereas the probe for *SORL1-fl* evenly labelled all Purkinje cells (PCs) as well as many cells in the granule cell layer (GCL), the probe for *SORL1-38b* showed a restricted signal mainly within the Purkinje cell layer and clearly absent from the GCL (Fig. 2d). Detailed inspection of the cerebellar sections tested for *SORL1* expression revealed a difference in the cellular distribution of the two probes (*p* < 0.0001). While the signal for *SORL1-fl* was predominantly (89%) located in the soma of PCs, *SORL1-38b* was only partially present in the PC soma (46%) with the majority of labelled transcripts located in the molecular layer where the dendritic tree of PCs extends (Fig. 2e, f).

These results pointed out PCs as the major source of production of E38b-containing *SORL1* transcripts in the cerebellum, and showed a regional difference between transcripts encoding SORLA-fl and SORLA-38b.

### Exon 38b determines neuronal dendritic targeting

**Fig. 3:**
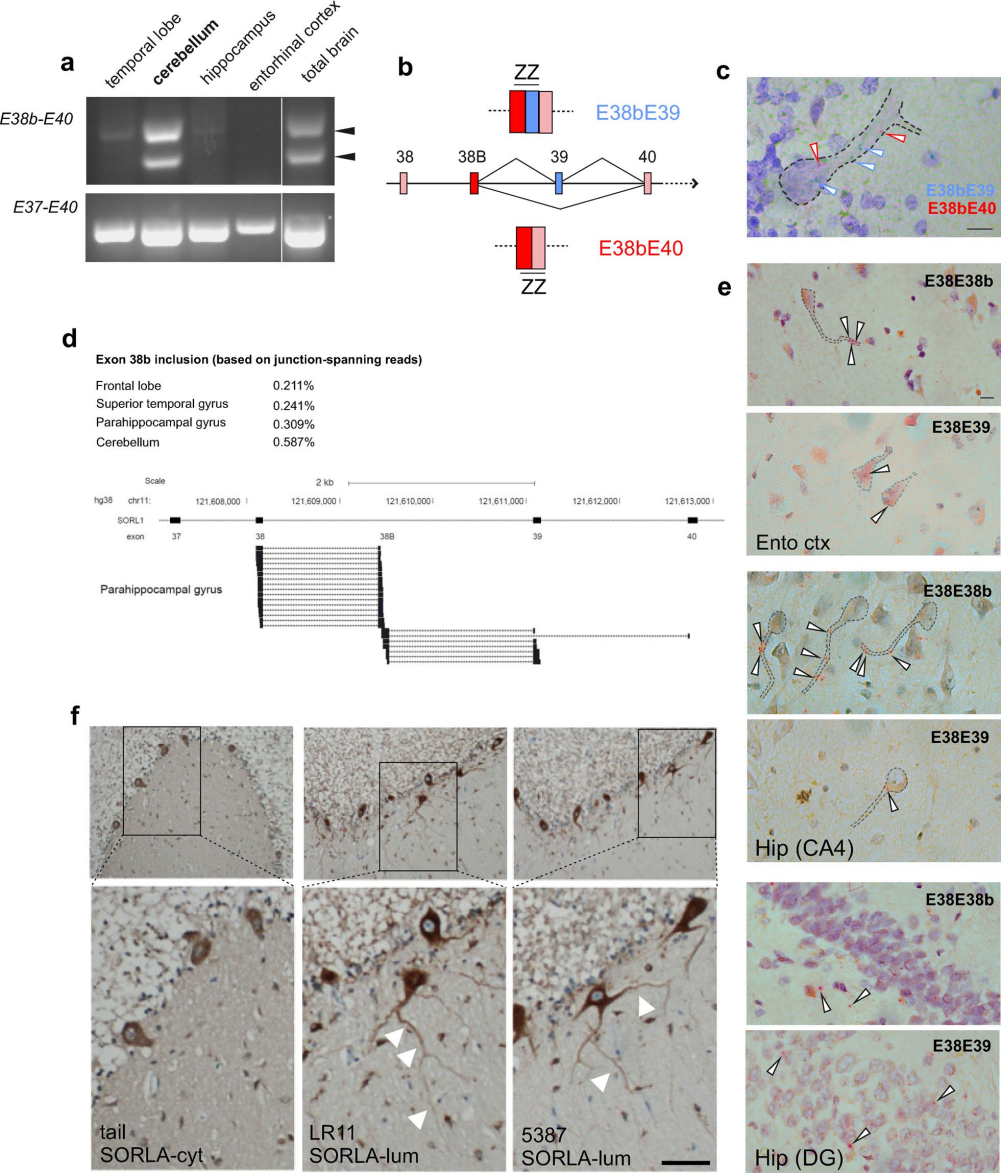
Alternative splicing of exon 38b determines soma-dendritic transport of *SORL1* transcripts. **a** RT-PCR using primers in exon 38b and 40 (E38b–E40) revealed the presence of the additional splicing event with skipping of exon 39 downstream the novel 38b cassette. **b** Schematic of BaseScope probe design for targeting *SORL1-38b* transcripts containing (E38bE39, blue) or excluding exon 39 (E38bE40, red). **c** Representative image of BaseScope Duplex using the probes E38bE39 (blue) and E38bE40 (red) shows that exon 39 is not responsible for soma retention. Scale bar, 20 μm. **d** Junction-spanning reads in human brain regions confirm the existence of two *SORL1-38b* transcripts from inclusion or exclusion of exon 39. Numbers represent the relative frequency of *SORL1-38b* reads among all *SORL1* reads (%). **e** BaseScope analysis using probes E38E38b and E38E39 shows the presence of extra-somatic *SORL1-38b* transcripts in different human brain areas. Signals for E38E39 that represent *SORL1-fl* are predominantly located in the neuronal soma. *Ento ctx* entorhinal cortex, *Hip* hippocampus, *DG* dentate gyrus. Scale bar, 50 μm. **f** Immunostaining of human cerebellum with an antibody raised against the cytoplasmic tail (SORLA-cyt) of SORLA-fl reveals that the full-length protein is mainly located to the soma of PCs. Staining with antibodies against the luminal part of SORLA (SORLA-lum; LR11, 5387) shows labeling of soma as well as dendrites of PCs (indicated with arrowheads)

As the 3′ UTR of transcripts is often involved in mRNA transportation in neurons [12, 43], we next applied a protocol for analyzing the 3′ end of the transcript by 3′ RACE PCR using RNA from human cerebellum. We found that both *SORL1-38b* and *SORL1-fl* end with exon 48 suggesting identical poly-adenylation for the two isoforms (Additional file 2: Fig. S2). We further examined the immediate 3′ region downstream of the stop-codon (introduced by inclusion of E38b) by RT-PCR using RNA from four different brain regions as well as whole brain. This analysis demonstrated the presence of two distinct E38b-containing transcripts, most notably in the cerebellum (Fig. 3a). Sequencing of these two PCR products identified a second alternative splice event where E38b can join directly to either the downstream exon 39 or to exon 40 by exclusion of exon 39.

Because transcripts for *SORL1-fl* contain exon 39 and mainly locate to the neuronal soma, we hypothesized a model where exon 39 would dictate somatic retention, and consequently account for the observed distribution of *SORL1-38b* transcripts split between soma and dendrites. A similar role for specific exons of BDNF mRNA has previously been demonstrated, showing how individual exons contain motifs responsible for soma retention or dendritic transport [3]. To investigate this, we designed two probes for BaseScope detection of *SORL1-38b* either including exon 39 (E38bE39) or skipping exon 39 (E38bE40) (Fig. 3b). We then applied the BaseScope Duplex assay for simultaneous visualization of both probes on the same cerebellum sample. In this experiment both probes gave signals within PC dendritic trees (Fig. 3c), indicating that skipping of exon 39 is not related to somatic localization of *SORL1-38b* transcripts, and suggesting that inclusion of E38b drives *SORL1* mRNA into dendrites.

Next, we analysed a number of human brain tissue RNA-seq datasets from available data repositories. By this approach we not only provide further evidence for the presence of *SORL1-38b* transcripts in all investigated samples, but also confirmed that skipping of exon 39 in the presence of E38b can occur in neurons in various brain regions, including the frontal lobe, hippocampal formations, and superior temporal gyrus (Fig. 3d). In agreement with our experimental findings, the highest number of *SORL1-38b* transcripts was observed for cerebellum, where it accounts for ~ 0.6% of all *SORL1* reads.

In situ analyses using probes E38E38b and E38E39 on human entorhinal cortex and hippocampal areas CA4 and dentate gyrus (DG) not only validated the expression of *SORL1-38b*, but also confirmed how most E38b-transcripts are located at the dendritic part of neurons opposed to somatic localization of *SORL1-fl* (positive for E38E39) in these brain regions (Fig. 3e).

We did several attempts to detect SORLA-38b expression in dendrites of cerebellar sections using our pAb-38b antibody. While we were able to see signal in PC soma and dendrites using this antibody (in line with the presence of *SORL1-38b transcripts)*, we experienced trouble with a background signal in the GCL that confounded the data. We therefore took an alternative approach to support a difference in cellular localization between truncated and SORLA-fl isoforms. Using an antibody that binds the cytoplasmic tail of SORLA, we found that SORLA-fl is almost exclusively located in the PC soma, whereas two different antibodies (IgGs LR11 and 5387), binding to epitopes in the extracellular receptor domain and therefore unable to distinguish between SORLA-fl and SORLA-38b, gave an additional strong signal in the dendritic tree of PCs that was only rarely seen in pictures from immunostainings using the tail antibody (Fig. 3f).

Together, our findings indicate that the *SORL1-38b* splice variant has a pronounced dendritic localization, likely underlining a yet undefined function. Further studies are necessary to elucidate the molecular drivers as well as the physiological conditions responsible for this dendritic targeting.

### The SORL1-38b transcript is downregulated in AD cerebellum

The cerebellum is historically considered a region of the brain showing little vulnerability for AD [6], although recent studies also link cerebellum to cognitive decline [16, 18]. Robust SORLA expression in cerebellum in AD has previously been observed [5, 13, 30, 38] despite decreased levels in disease-affected regions including frontal cortex and hippocampus [1, 14, 30]. However, these previous studies did not take into account that alternative isoforms of *SORL1* may be altered. As dendritic and synaptic connectivity loss is one of the driving events occurring in AD, we next asked if *SORL1-38b* levels are affected in the cerebellum of AD patients.

**Fig. 4:**
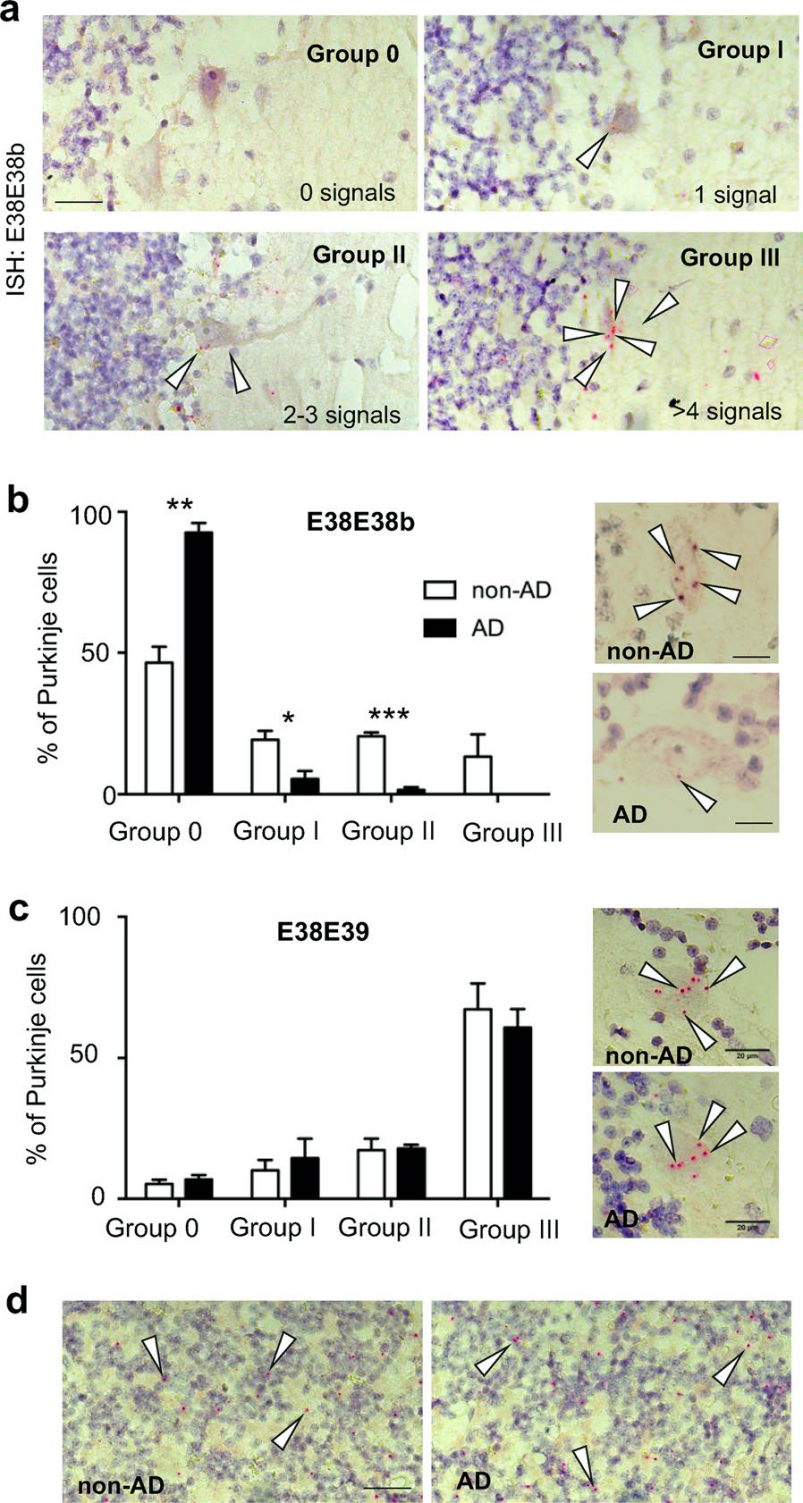
Reduced expression of the alternatively spliced *SORL1-38b* transcript in cerebellum of AD patients. **a** Representative images of ISH in human cerebella from non-AD individuals using E38E38b probe for detection of *SORL1-38b* transcripts. Cells were grouped in 4 different groups according to copy-number of mRNAs: group 0 (0 signals), group I (1 signal), group II (2–3 signals), and group III (> 4 signals). Scale bar, 50 μm. **b** Quantification of BaseScope signal in groups of PCs with E38E38b probe confirms significant reduction of *SORL1-38b* transcripts in the cerebellum from AD patients (N = 3, n = 1006; non-AD; N = 3, n = 1444). Scale bar, 20 μm. *p<0.05, **p<0.01, ***p<0.001. **c** Quantification of BaseScope readouts indicates no significant differences of E38E39 probe signal in AD cerebella compared to non-AD controls (non-AD, N = 3, n = 2936 cells in total; AD; N = 3, n = 2768 cells in total). Scale bar, 20 μm. **d** Representative images of ISH signal using E38E39 probe show that *SORL1-fl* is similarly expressed in GCs in non-AD and AD cerebella. Scale bar, 50 μm

As expected, tissue sections from cerebellum of AD and non-AD individuals showed no gross abnormalities despite disease status. To ensure comparable RNA quality between AD and non-AD samples, we hybridized the cerebellar sections with a probe targeting the human *PPIB* gene, indicating no general RNA decay in these cerebellar AD samples (Additional file 3: Fig. S3). We next performed a quantitative analysis of the expression of *SORL1-38b* transcripts in PC soma by BaseScope assay by applying probe E38E38b to tissue sections from 3 AD and 3 non-AD individuals (sample data listed in Table 1), and quantified the levels of transcripts. As indication of the expression level, we could group the PCs according to the copy number of E38b-containing transcripts expressed by PCs, and we classified into four groups according to the signal detected per cell soma (Fig. 4a). Surprisingly, we observed a markedly reduced expression of the *SORL1-38b* isoform in all three AD samples (Fig. 4b).

Detailed quantification based on the signal in the soma revealed that 46.6% of all measured PCs (n = 1444) were negative (group 0) by the E38E38b ISH analysis in non-AD cerebella, and the remaining 53.4% positive PCs distributed equally between groups I-II-III (19.7% group I; 20.5% group II; 13.2% group III). However, in cerebella from AD patients, 93.0% of all PCs showed a lack of *SORL1-38b* expression (*p* < 0.001 compared to non-AD distribution in group 0), accompanied by a significant reduction in the number of PCs corresponding to groups I (5.5%; *p* = 0.0177) and II (1.5%; *p* = 0.0002) with no cells assigned to group III (Fig. 4b).

In parallel, we tested the same brains to detect *SORL1-fl* transcripts. In line with previous reports that showed robust SORLA expression in AD cerebellum [5, 30], we did also not see any difference for *SORL1-fl* levels between AD and non-demented controls (Fig. 4c). Quantification of the hybridization signal for the E38E39 probe showed that the majority of PCs (67.3%) expresses high levels of *SORL1-fl* transcript (group III), and that none of the four groups were affected by AD (group 0, *p* = 0.4197; group I, *p* = 0.4674; group II, *p* = 0.8955; group III, *p* = 0.5474) (Fig. 4c). Similarly, we did not observe any decrease in the signal for *SORL1-fl* in the GCL (Fig. 4d). These findings confirmed *SORL1-fl* is abundantly and robustly expressed in the human cerebellum, and its expression is not affected by AD in this brain region.

### Replication analysis of transcript levels

**Fig. 5:**
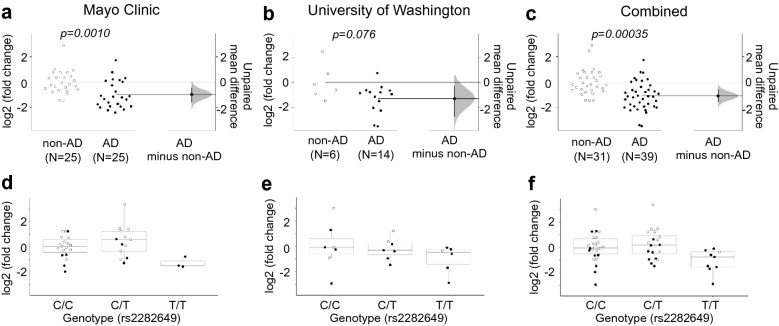
Meta-analysis of *SORL1-38b* expression in non-AD and AD cerebellum. **a** TaqMan qPCR assay using a probe designed for detection of *SORL1-38b* transcripts shows a 2.0-fold reduction in expression levels in AD cerebellum compared to non-AD controls from a Mayo Clinic cohort (non-AD; N = 25, AD; N = 25). **b** qPCR analysis on non-AD and AD cerebellum from UW cohort confirms reduction of *SORL1-38b* in AD samples (non-AD; N = 6; AD; N = 14). **c** Meta-analysis including expression levels of *SORL1-38b* in non-AD versus AD cerebella from Mayo Clinic and UW cohorts. **d**–**f** Relative expression of *SORL1-38b* to the genotype of SNP24 located in the 3′ haplotype of *SORL1* (rs2282649) in Mayo Clinic (**d**; non-AD; N = 23, AD; N = 12), UW cohort (**e**; non-AD; N = 6; AD; N = 14), and meta-analysis including data from both cohorts (**f**)

To replicate the finding of lowered *SORL1-38b* expression in brains from AD patients, we used Taqman qPCR to quantify *SORL1-38b* levels in AD versus non-AD brains in two additional independent cohorts. First, we investigated 25 AD and 25 non-AD post-mortem cerebella samples identifying a mean 2.0-fold down-regulation of *SORL1-38b* transcript levels in AD cerebellum compared to controls (*p* = 0.0010) (Fig. 5a). Of notice, this cohort was previously analyzed and showed no reduction of *SORL1-fl* in AD patients compared to controls [5], demonstrating again that the two *SORL1* isoforms are differently affected by AD. Consistent with this, a qPCR analysis of 14 AD and 6 non-AD cerebella samples identified a mean 2.4-fold down-regulation of *SORL1-38b* transcripts in AD brains compared to non-AD brains (*p* = 0.076) and no change in *SORL1-fl* (Fig. 5b). Quantification of the PC marker Calbindin relative to the reference genes showed no differences between AD and non-AD samples from the UW cohort (ΔΔCt = − 0.08; *p* = 0.869). A meta analysis of the two cohorts (in total 39 AD; 31 non-AD) found a mean 2.1-fold decrease of *SORL1-38b* in AD (p_meta_ = 0.00035) (Fig. 5c).

### Transcript levels stratified by SNP24

The first association study focusing on *SORL1*, carried out by Rogaeva and coworkers in 2007 [36], identified an association with AD for two different haplotypes in *SORL1*, more specifically a 5′ risk-haplotype and a 3′ risk-haplotype in the gene [36]. While the association with 5′ risk-haplotype has been linked to reduced BDNF-induced *SORL1* activity [49], the underlying reason for the 3′risk haplotype association remains unclear. In 2019, a large GWAS of AD identified a single risk locus in *SORL1* with lead SNP rs11218343 [20]. The locus spans around 68 kb (from rs7946599 to rs75439772) and includes E38b as well as the 3′ haplotype [20] (Additional file 4: Fig. S4a). One of the SNPs tagging the 3′ haplotype is rs2282649, labelled SNP24 in the Rogaeva study [36]. Because of the association previously reported and the genomic position of SNP24 close to E38b (around 1200 bp), we speculated if rs2282649/SNP24 was associated with AD, and if SNP24 genotypes correlated with *SORL1-38b* levels. Out of the 50 Mayo cerebellum samples, 35 samples had genotypes available for rs2282649/SNP24 from whole-genome sequencing in the AMP-AD knowledge portal. The samples from UW were genotyped for SNP24 and selected to enrich for individuals with the T/T genotype.

In the Mayo subset (12 AD and 23 non-AD) the SNP24 allele frequency of the risk allele (T-allele) was higher among AD samples (41.7%) compared to non-AD samples (19.6%) (*p* = 0.09), supporting a trend for association of this variant with AD in this small sample. Because samples from the UW cohort were selected for qPCR based on their SNP24 genotype, allele frequencies are not representative for AD cases and controls, and they were not included in the association analysis with disease status.

Stratifying the Mayo and the UW samples according to SNP24 genotypes revealed a decreasing tendency in *SORL1-38b* levels in carriers of the risk genotype T/T of SNP24 (rs2282649) (Fig. 5d, e) (*p*_*meta*_ = 0.14, analysis adjusted for AD status) (Fig. 5f). To determine if SNP24 is a true eQTL for *SORL1-38b* levels, a larger number of samples is needed, with both genotypes *and* E38b transcript levels available.

To test if any stronger eQTLs for 38b existed in the *SORL1* locus, genetic variants in *SORL1* as well as up- and downstream were downloaded from the AMP-AD knowledge portal for the 35 Mayo samples. Apart from a small cluster of SNPs in the *SORL1* promoter, which was to be expected, no other strong eQTL could be identified for E38b-transcript levels within the *SORL1* locus (Additional file 4: Fig. S4b). Future studies with a larger number of individuals, including also carriers of the GWAS index SNPs, could reveal if the mechanism underlying common-variant association of *SORL1* with AD involves the E38b transcript.

## Discussion

We have previously shown how a *SORL1* transcript that differs from full-length is generated by an alternative transcriptional start site [5], but *SORL1-38b* is to our knowledge the first alternatively spliced isoform of the AD gene *SORL1* that has been documented. Interestingly, our findings showed that the inclusion of E38b in transcripts primes mRNA translocation targeting *SORL1-38b* to extra-somatic compartments in neurons. Such asymmetrical distribution of mRNA may be more thermodynamically efficient than transporting proteins as fewer mRNA molecules need to be mobilized [8]. Neurons in particular rely on mRNA translocation as a mechanism to coordinate regional and temporal changes in protein levels in both axonal and dendritic compartments, as well as by local protein synthesis to affect synaptic plasticity of individual synapses [7].

For our studies, we focused on the cerebellum showing the highest relative expression of *SORL1-38b*, like for *SORL1-fl*. Also, we confirmed both the expression of this isoform and its dendritic localization in neurons of hippocampus and cortical regions that are affected by AD. This suggests the cerebellum as a model system to study *SORL1-38b* biology, allowing the translation of our findings to other brain areas affected by AD pathology. The high cerebellar expression may reflect the fact that Purkinje neurons have the most elaborate dendritic trees among neurons in the brain [42], with a huge number of dendritic spines that could be particularly dependent on *SORL1* splicing. The role of *SORL1-38b* is not clear, but it may act similarly to protocadherins where AS has been reported to influence self-avoidance of dendrites in cerebellar PC [23]. Accordingly, *SORL1-38b* could play an important role to provide synapse and neuronal identity.

Here, we provide evidence that *SORL1-38b* is decreased in the cerebellum of patients with AD, using samples from three independent cohorts. While *SORL1-fl* is not decreased in cerebellum of AD patients [2, 5, 30], we found a significant decrease of *SORL1-38b* in AD patients from all three cohorts. This is interesting as cerebellum, which is usually described as unaffected in AD, recently has gained attention as playing a role in cognitive decline [18], with evidence showing that network-based degeneration in AD extends to the cerebellum [16]. Although AS has previously been studied in relation to AD [21, 37], this is the first description of a *SORL1* isoform affected in the cerebellum of AD patients.

The specific decrease of *SORL1-38b* levels in AD suggested a unique difference in either production or degradation between this isoform and *SORL1-fl*. An AD-dependent change of the splicing machinery necessary for E38b inclusion may explain differences in the production of the two isoforms. Alternatively, a decrease in the dendritic machinery responsible for transporting the mRNA or being related to previously reported synaptic and dendritic loss in AD including loss of synapses of PCs in AD [25, 26] could explain the observed decrease. However, any mechanism where *SORL1-38b* loss is a consequence of its localization into dendrites/synapses would seem to require a feedback mechanism in order to additionally explain the observed decrease of *SORL1-38b* in the neuronal soma by ISH. Interestingly, the most severe reduction of *SORL1-38b* in AD cerebella was found in the NBB cohort, only including samples at advanced stages compared to UW and Mayo cohorts. This could reflect a positive correlation between severity of E38b-transcripts downregulation and stage of AD.

If the identified reduction of E38b is a consequence of synaptic loss due to AD, our findings may also be relevant for other neurodegenerative disorders like Parkinson’s disease and frontotemporal dementia. It would therefore be of interest for future studies to determine *SORL1-38b* levels also for non-AD degenerative diseases, as well as looking further into the possible association between *SORL1-38b* and SNP24 that is specifically reported to associate with AD [36].

Translation of transcripts including E38b leads to production of a truncated SORLA isoform lacking four 3Fn-domains as well as transmembrane and cytoplasmic domains that are all present in SORLA-fl. This suggests different functions of the two isoforms, in line with multiple studies reporting that locally synthesized proteins are most often structurally and functionally distinct from proteins present at the same cellular site but being transported to their destination [8]. Usually, transcripts containing a premature stop codon (PSC) are targets of the nonsense mediated decay (NMD) pathway. However, it has been previously shown that a human specific splice variant of the *Survival Motor Neuron 1* (*SMN1*) gene holding a PSC, after introduction of a novel exon, is able to escape NMD and be locally translated in neuronal terminals [40]. Similarly, NMD degradation of *SORL1-38b* may be prevented by trafficking the transcript to dendrites where the truncated SORLA-38b protein is produced.

SORLA-fl is known to regulate sorting of a number of cargo proteins including APP and the Amyloid-β peptide [1, 9], in a process that requires complex formation between the SORLA tail domain and cytosolic trafficking factors, most notably the retromer complex [15]. This activity determines sorting of SORLA and its cargo in Golgi and endosomal compartments of the neuronal soma. Here, we were puzzled why SORLA-38b is not merely being secreted in the absence of the transmembrane anchor, as a construct encoding the longer extracellular region of SORLA containing all six 3Fn-domains become efficiently secreted [39]. Our experiment to determine if the presence of the E38b-encoding 22 amino acids was responsible for this cellular retention showed that the E38b-encoded peptide was not responsible for the lack of secretion. Subsequent studies revealed that the ability to leave the cell is determined by the presence of the fifth 3Fn-domain in the SORLA ectodomain, likely reflecting conformational rearrangements of the SORLA extracellular part in the absence of this domain. This strongly support our hypothesis that SORLA-38b has its own cellular function unrelated to SORLA-fl.

The exact function of the shorter SORLA-38b isoform is not clear, but it is very tempting to speculate a role related to synaptic plasticity, which is the most frequent function associated with localized protein synthesis in dendrites [45]. The study of the function of SORLA-38b is complicated as it is not expressed in rodents. We attempted to differentiate neurons from human induced pluripotent stem cells, however the applied protocol previously shown to provide a model for studying the function of SORLA-fl [22] unfortunately failed to induce sufficient levels of *SORL1-38b* expression (determined by qPCR; data not shown). This observation is in line with our present findings where only a subset of neurons expresses the alternatively spliced isoform. We suggest future studies should apply protocols for differentiation into PCs [47, 48] to increase the possibility that the induced neurons will express sufficient SORLA-38b to allow for functional studies.

SORLA has previously been speculated to act as an adhesion protein, because 3Fn-domains are often present in adhesion molecules, including protocadherins, immunoglobulin superfamily and contactins [4, 10]. Interestingly, inclusion of an alternative exon in *CNTN4* (encoding Contactin-4) introduces a novel stop codon and generates a truncated protein with deletion of several 3Fn-domains [50] similarly to what we here describe for SORLA-38b. Detection of the truncated form of Contactin-4 in human brain samples confirmed the production of a stable isoform. AS of proteins involved in cellular adhesion has been reported to generate extensive diversity for neuronal intercellular recognition, often in a cell type specific way. Such splice-dependent interactions play fundamental roles in neural circuit establishment and maintenance, including repulsion and attraction allowing neurites to form specific synapses [34]. Local synthesis of SORLA-38b in dendrites would be in perfect agreement with such a function in cellular contact formation at the synapse, and may also relate to our observation that expression of SORLA-38b is restricted to a subset of neurons—providing neuronal identity—in comparison to SORLA-fl with a broader expression profile and function.

In conclusion, for the characterization of the lowly expressed *SORL1-38b* we have established several methods including the development of a specific antibody, the application of E38b-specific probes for ISH and the optimization of qPCR assays. Altogether, these made the identification of an otherwise hardly detectable splice isoform possible, providing valuable information about *SORL1* transcriptomic profile.

Although the study revealed novel findings about *SORL1*, a few limitations need to be mentioned. First, the low expression level of *SORL1-38b* challenged the detection of this transcript in other brain regions than the cerebellum. Thus, the implementation of more sensitive techniques, as well as the optimization of established protocols, will aim at confirming our data in non-cerebellar areas. Second, the small sample size of the cerebellum cohorts limited the analysis of *SORL1-38b* expression in relation to the risk T/T (SNP24) genotype. Future studies including larger cohorts will help clarifying how *SORL1-38b* associates with SNP24.

## Conclusions

Here, we provide the first detailed characterization of an alternatively spliced transcript of the *SORL1* gene. Our results highlight two major novel findings in *SORL1* research: the dendritic localization of *SORL1-38b* transcripts and the specific reduction of E38b levels in cerebellum from AD patients despite *SORL1-fl* transcripts being unaltered. Altogether, these data add another layer of complexity to the understanding of *SORL1* biology.

## Supplementary Information

**Additional file 1. Figure S1**: SORLA-38b is retained intracellularly in HEK cells. **a** Substitutions of cysteine to alanine at position 1 and 13 of the unique tail encoded by E38b do not alter the secretion of SORLA-38b in the medium. Non transf non transfected cells. **b** WB analysis of lysates and media from CHO cells transiently transfected with SORLA-fl, SORLA variants where stop mutations were introduced at the boundary following each 3Fn domain, or a SORLA variant lacking the cytoplasmic tail (SORLA-ΔCD). Notably, constructs lacking the 3Fn5 are most strongly retained intracellularly (indicated with arrowheads).

**Additional file 2. Figure S2**: Analysis of the 3′ end of *SORL1-38b*. Agarose gel showing 3′ RACE products for *SORL1-fl* and *SORL1-38b*. Bands indicated with arrows have been sequenced.

**Additional file 3. Figure S3**: Control of RNA quality in non-AD and AD cerebella. Samples hybridization with PPIB probe showed no distinct difference in the signal from non-AD compared to AD cerebella.

**Additional file 4. Figure S4**: Analysis of genetic variants affecting the expression of E38b. **a** Representation of the boundaries of the 3′ end locus in *SORL1* including localization of E38b. **b** eQTL mapping of *SORL1* gene did not show significant association with E38b expression (SNP24: rs2282649).

## Data Availability

The results published here are in part based on data obtained from the AMP-AD Knowledge Portal (https://adknowledgeportal.synapse.org/). Whole genome-sequence data for 35 of the Mayo Clinic samples is available via the AD Knowledge Portal (https://adknowledgeportal.synapse.org, 10.7303/syn2580853).
