# Supplementary figures and images for: Expression of an alternatively spliced variant of SORL1 in neuronal dendrites is decreased in patients with Alzheimer’s disease
Source: Acta Neuropathol Commun. 2021 Mar 16;9:43. doi: 10.1186/s40478-021-01140-7 (PMC7962264; doi:10.1186/s40478-021-01140-7)

Monti *et al*  
Figure S1

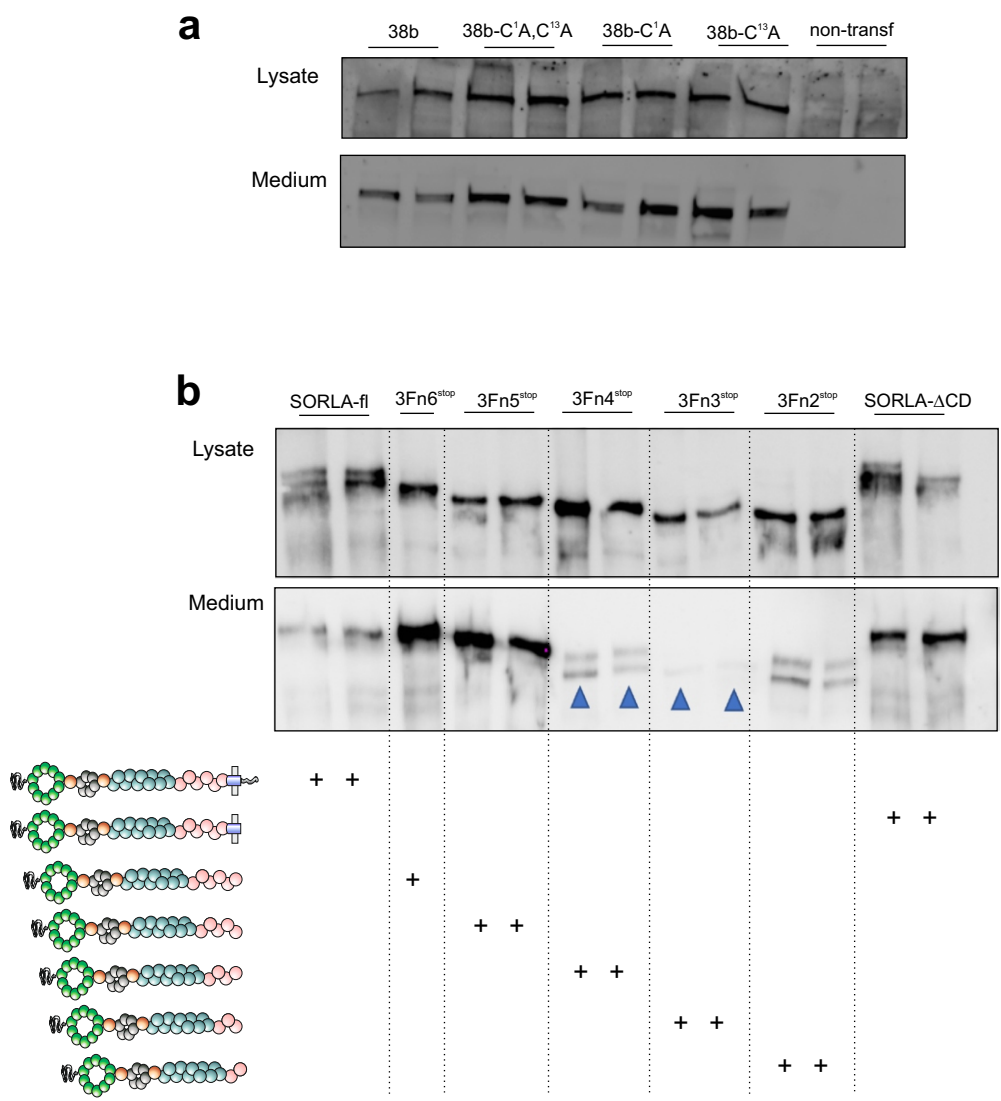

Supplement: Supplementary file 1 — Additional file 1. Figure S1: SORLA-38b is retained intracellularly in HEK cells. a Substitutions of cysteine to alanine at position 1 and 13 of the unique tail encoded by E38b do not alter the secretion of SORLA-38b in the medium. Non transf non transfected cells. b WB analysis of lysates and media from CHO cells transiently transfected with SORLA-fl, SORLA variants where stop mutations were introduced at the boundary following each 3Fn domain, or a SORLA variant lacking the cytoplasmic tail (SORLA-ΔCD). Notably, constructs lacking the 3Fn5 are most strongly retained intracellularly (indicated with arrowheads). [file 40478_2021_1140_MOESM1_ESM.pdf]

Monti *et al*  
Figure S2

### 3 RACE

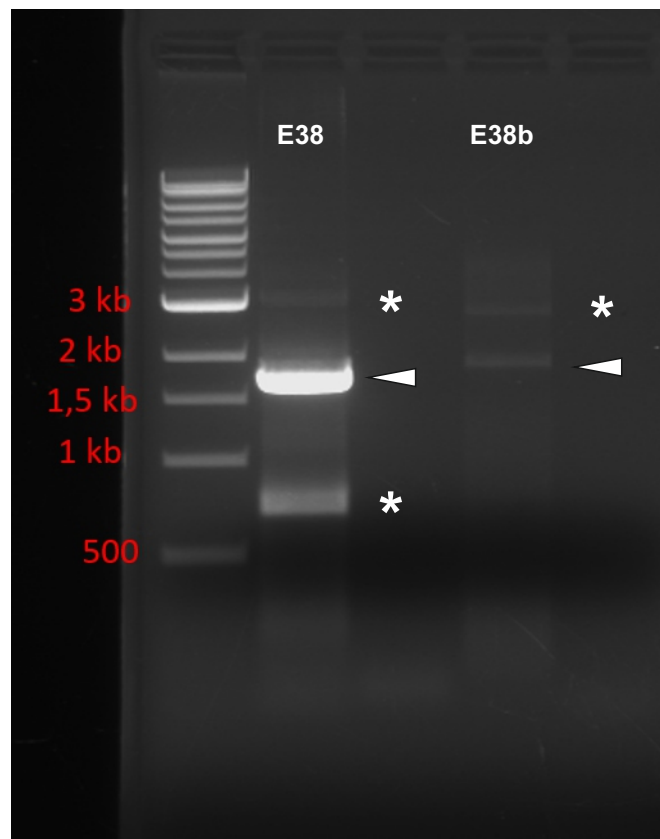

Supplement: Supplementary file 2 — Additional file 2. Figure S2: Analysis of the 3′ end of SORL1-38b. Agarose gel showing 3′ RACE products for SORL1-fl and SORL1-38b. Bands indicated with arrows have been sequenced. [file 40478_2021_1140_MOESM2_ESM.pdf]

Monti *et al*  
Figure S3

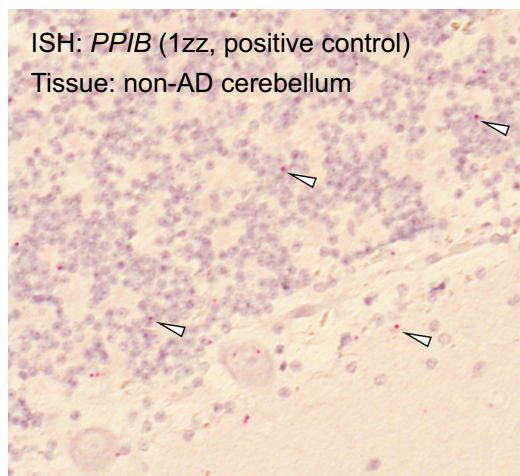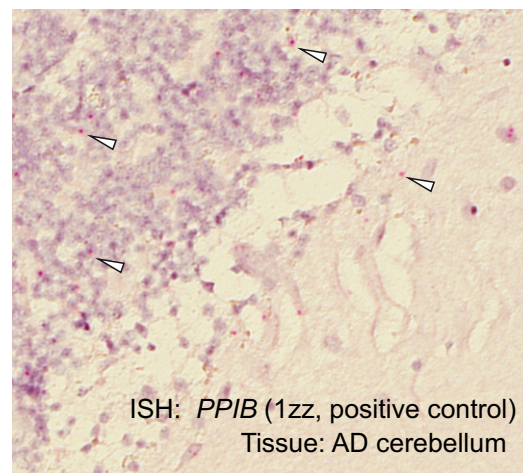

Supplement: Supplementary file 3 — Additional file 3. Figure S3: Control of RNA quality in non-AD and AD cerebella. Samples hybridization with PPIB probe showed no distinct difference in the signal from non-AD compared to AD cerebella. [file 40478_2021_1140_MOESM3_ESM.pdf]

Monti *et al*  
Figure S4

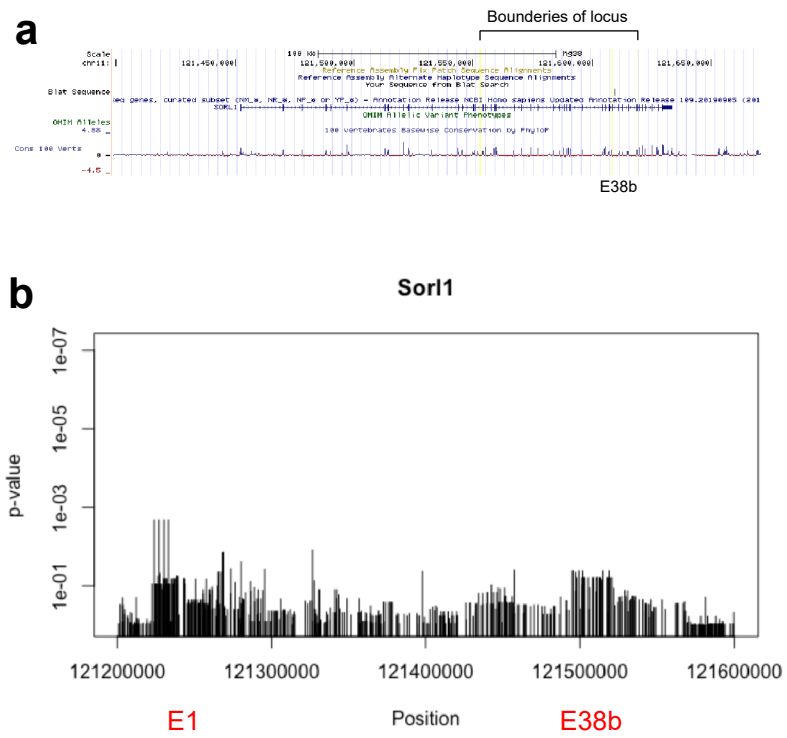

Supplement: Supplementary file 4 — Additional file 4. Figure S4: Analysis of genetic variants affecting the expression of E38b. a Representation of the boundaries of the 3′ end locus in SORL1 including localization of E38b. b eQTL mapping of SORL1 gene did not show significant association with E38b expression (SNP24: rs2282649). [file 40478_2021_1140_MOESM4_ESM.pdf]
